# Automated face recognition of rhesus macaques

**DOI:** 10.1016/j.jneumeth.2017.07.020

**Published:** 2018-04-15

**Authors:** Claire L. Witham

**Affiliations:** a Institute of Neuroscience, Newcastle University, Newcastle-upon-Tyne, UK; b Centre for Macaques, Medical Research Council, UK

**Keywords:** LBP, local binary patterns, NN, nearest neighbor, LDA, local discriminant analysis, SVM, support vector machine, Monkey, Face detection, Face recognition, Computer vision

## Abstract

**Background:**

Rhesus macaques are widely used in biomedical research. Automated behavior monitoring can be useful in various fields (including neuroscience), as well as having applications to animal welfare but current technology lags behind that developed for other species. One difficulty facing developers is the reliable identification of individual macaques within a group especially as pair- and group-housing of macaques becomes standard. Current published methods require either implantation or wearing of a tracking device.

**New method:**

I present face recognition, in combination with face detection, as a method to non-invasively identify individual rhesus macaques in videos. The face recognition method utilizes local-binary patterns in combination with a local discriminant classification algorithm.

**Results:**

A classification accuracy of between 90 and 96% was achieved for four different groups. Group size, number of training images and challenging image conditions such as high contrast all had an impact on classification accuracy. I demonstrate that these methods can be applied in real time using standard affordable hardware and a potential application to studies of social structure.

**Comparison with existing method(s):**

Face recognition methods have been reported for humans and other primate species such as chimpanzees but not rhesus macaques. The classification accuracy with this method is comparable to that for chimpanzees. Face recognition has the advantage over other methods for identifying rhesus macaques such as tags and collars of being non-invasive.

**Conclusions:**

This is the first reported method for face recognition of rhesus macaques, has high classification accuracy and can be implemented in real time.

## Highlights

• First study showing automated face recognition of rhesus macaques.
• High levels of classification accuracy.
• Methods can be implanted in real time using standard hardware.
• Potential application to social analysis demonstrated.

## Graphical abstract

**Figure:**
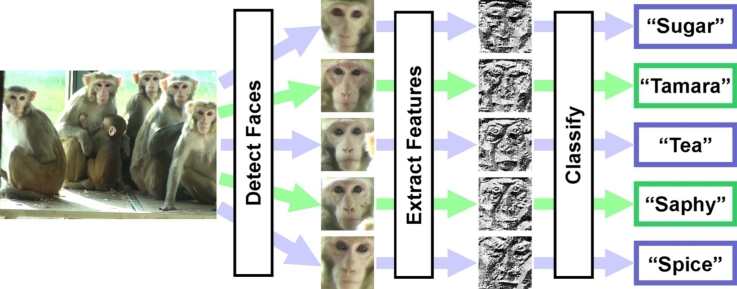

## 1 Introduction

Automated methods for monitoring behavior of laboratory animals such as rodents and zebrafish are becoming widespread (Bains et al., 2016, Nema et al., 2016, Steele et al., 2007). They allow the monitoring of behavior effects in response to experimental manipulations such as drugs, lesions, genetic modifications and disease. Concurrently similar automated behavior systems are being developed for monitoring health and welfare in a range of species including farm and laboratory animals (Roughan et al., 2009, Rushen et al., 2012). Rhesus macaques are one of the most common non-human primate species used in biomedical research including neuroscience research but to date the use of automated systems with non-human primates has been limited.

A major challenge in measuring the behavior of any group-housed animal is to reliably identify an individual animal. With macaques this is not an issue if the animals are singly-housed but welfare concerns are driving a move towards pair- and group-housing of non-human primates in many countries. One solution is to add a tracking device to each animal; for example these could be colored jackets (Rose et al., 2012) or collars (Ballesta et al., 2014) in combination with video monitoring or electronic devices such as RFID tags (Maddali et al., 2013). These require regular handling of the animals and it is not currently known how the use of jackets and collars affects the behavior of the animals (personal observations with rhesus macaques suggest that the use of jackets can drastically reduce social behaviors such as grooming).

Another solution is to use biometric identification based on the distinguishing visual characteristics of that species (e.g. coat pattern; Kühl and Burghardt, 2016). This has the advantage of being non-invasive. Rhesus macaques, in common with many primate species, do not have obvious individually identifiable features but the macaques themselves are capable of recognizing conspecifics by their faces (Parr et al., 2000). Face recognition technology has already been used in several non-human primate species including guenons (Allen and Higham, 2015), chimpanzees (Freytag et al., 2016) and gorillas (Loos, 2012) but not rhesus macaques. Freytag et al. (2016) achieved success rates of over 90% with images of captive chimpanzees.

Face recognition technology was originally developed for use with humans and is becoming commonplace in daily life. Uses include automatic passport gates at airports, tagging of faces in photos on Facebook and use of facial image to unlock smart phones. Many of the early techniques focused on either reducing the dimensionality of the facial image or on extracting a particular feature from the image and then on classifying this output. Some of these methods for face recognition include EigenFaces (based on principal component analysis) and FisherFaces (based on linear discriminant analysis; Belhumeur et al., 1997). Some of the main challenges facing any face recognition system are coping with changes in light intensity and pose. A method based on local binary patterns (Ahonen et al., 2006) has been shown to be relatively robust to changes in light intensity. Most recently deep learning techniques have been applied to face recognition with a high level of success (Freytag et al., 2016 for chimpanzees; Parkhi et al., 2015 for humans).

Here I describe methods for performing face detection and recognition on images and videos of rhesus macaques. The methods were developed under the constraints that training and processing should be possible on a standard PC and that there should be the possibility of using the methods for online (real-time) analysis. To make it easier to integrate into existing setups I have focused on the more established face recognition methods rather than the latest methods. These methods are validated on a model image set of 34 adult macaques and are tested under a range of challenging conditions. I demonstrate an application of these methods to monitoring social relationships in group-housed macaques. Finally I show that these methods can be applied in real time at frame rates of up to 15 frames per second using a standard laptop and an USB camera.

## 2 Materials and methods

### 2.1 Animals

All methods presented were developed using group-housed rhesus macaques (*Macaca mulatta*) at a breeding facility. The monkeys were housed in groups of 6–25 (ages in the range 0–20 years) in large indoor enclosures (enclosures consist of two separate areas; the first has dimensions 8.04 m length × 3.35 m width × 2.8 m height and the second has dimensions 6.12 m length × 1.5 m width × 2.8 m height). The enclosures have high levels of enrichment and access to natural light The housing exceed the national guidelines (UK Home Office Code of Practice) and all necessary approvals for this study (including ethical through the local Animal Welfare and Ethics Review Board) were given. As part of routine husbandry at the colony the monkeys are tattooed with an abbreviation of their ID on their chests. This is done under ketamine sedation at the approximate age of 12 months and allows care staff to identify individuals.

### 2.2 Video

High definition video footage was collected using a camcorder (Sony HDR-SR12E). For each group the camera was set up on an internal window overlooking the main enclosure and aimed at the rear of the enclosure (this area included a large bay window where the monkeys liked to sit and socialize with each other). The videos were converted from AVCHD format to the more common MPEG-4 format (settings: 1920 × 1080 size, 20 frames per second frame rate and 4000 kbps bit rate) using Aiseesoft HD Video Converter (www.aiseesoft.com). Each video was annotated with the date and group information.

### 2.3 Facial image sets

**Fig. 1:**
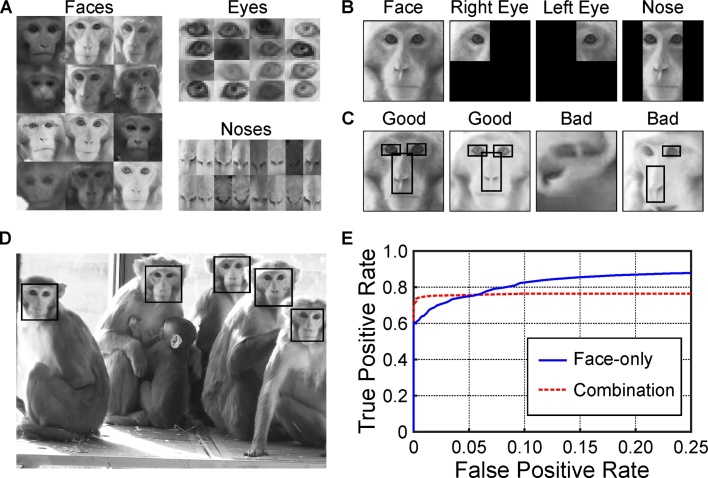
Face detection. A, examples of images used to train face, eye and nose cascade classifiers. B, regions of face run through eye and nose cascade classifiers. C, images accepted (Good) or rejected (Bad) by alignment stage. D, face detection run on a single video still, black boxes indicate detected faces. E, receiver operating characteristic curve for face detection for face-only and combination detectors.

Three main image sets were used in this study. The first image set (detection training image set) consisted of images of macaque faces (1189 images), eyes (385 images) and noses (451 images) manually cropped from videos stills featuring many different monkeys (cropping was performed using Adobe Lightroom; www.adobe.com). This image set included images of both sexes and a wide range of ages from new born to fully adult. The set was used to train the cascade classifiers used for face and feature detection (Section 2.4). Examples of the images used in the set are shown in Fig. 1A. The second image set (detection testing image set) consisted of a range of 428 images containing between 0 and 4 faces and representing multiple different views. This image set included frames from the videos recorded with the camcorder, frames taken from Point Grey USB cameras (see Section 2.7) and photos taken with a Canon camera (Canon 7D); the idea being to test the performance across a range of different setups. The number of faces and the location of each face within the frame was identified by a human observer to produce a ground-truth dataset for testing face detection performance.

The third image set (recognition image set) is based on images extracted from four different breeding groups using the face detection techniques discussed in Section 2.4. This produced over 100,000 facial images of macaques (each video was processed at a rate of 2 frames per second), collected from videos taken on multiple days over a one year period. These images were manually sorted according to the identity of the monkey and a subset of images per monkeys were further sorted by the quality of the facial image. From this a smaller image set for testing face recognition was constructed that contained 50 well-aligned images and 100 random images from each of 34 adult monkeys (30 female and 4 male; age range 3–20 years). Only adults were included as the facial features of infants changed substantially over the one year filming period. In addition further sets of 10 images were selected from four categories that should challenge the face recognition system. These categories were high-contrast, facial expressions, partial occlusion and rotation (this was done for each monkey where there was sufficient images of this class).

### 2.4 Face detection

#### 2.4.1 Training

Face detection is a prerequisite for most forms of face recognition. A face detector was created based on the cascade classifier approach developed by Viola and Jones (2001. The classifier contains 13 stages and is based on local binary pattern features (see Section 2.5 for more details on local binary patterns). Each stage consists of a single weak classifier and is automatically selected during the training procedure. It was trained using images from the detection image set and a set of negative images (2958 images including images of the enclosure and images of other views of the monkeys). This classifier had 13 stages, an object training size of 24 × 24 pixels, a hit rate of 0.995 and a false alarm rate of 0.5.

This detector was effective but produced high levels of false positives. The approach was refined by training two further feature detectors; one for a single eye (20 stages; object training size of 17 × 10 pixels) and one for a nose (17 stages; object training size of 20 × 40 pixels). These were applied as shown in Fig. 1B. Any faces detected in the original video still are cropped and resized to 100 × 100 pixels. The eye detector is then run on the upper left and right quadrants to look for the right and left eyes respectively and the nose detector is run on the central column. The detected face is only accepted as “Good” if it contains all three features (Fig. 1C).

#### 2.4.2 Processing videos

To provide training images for testing face recognition (recognition image set; see Section 2.3) face detection was run on videos at a rate of 2 frames per second. Face detection was run on each frame using the cascade object detector function in Matlab (with a merge threshold of 8 and a scale factor of 1). Each potential facial image was cropped and resized to 100 × 100 pixels and then eyes and nose detection run on the cropped image (also using the cascade object detector function; see Section 2.4.1 for more details). Facial images that met the criteria were saved as jpeg files along with a processed version of the original video still to allow for easy sorting.

### 2.5 Face recognition

#### 2.5.1 Local binary patterns

**Fig. 2:**
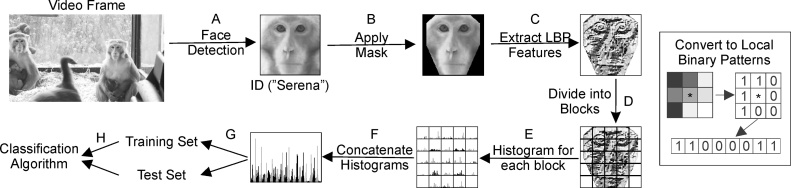
Diagram of face recognition stages. Inset, calculating the local binary pattern for a single pixel (*).

A local binary pattern approach was taken for face recognition. Local binary patterns are easy and quick to calculate, are robust to certain changes in illumination and are an established method for face recognition in humans (Ahonen et al., 2006). For the most basic local binary pattern the intensity of each pixel with its 8 surrounding pixels (as shown in inset in Fig. 2) and assigning a 1 if the central pixel is lighter than the surrounding pixel and 0 if it is darker. An 8‐bit binary code is then assigned to the central pixel (this can be converted to a number between 0 and 255). Some patterns are more informative for face recognition than others. A uniform local binary pattern is one where there are only a couple of changes from 0 to 1 (or vice versa) in the pattern and usually denotes a feature such as an edge or corner (Ahonen et al., 2006).

#### 2.5.2 Face recognition stages

For face recognition there are two stages: training and testing. In both cases the facial images found by the face detection procedure in Section 2.4 were treated in the same way as illustrated in Fig. 2. First the detected images were resized to 100 × 100 pixels, converted to grayscale (A) and manually sorted by identity (in Fig. 2 the monkey shown is named Serena). A mask was then applied to focus on just the facial area (B) and then the image was converted to local binary patterns (C). The image was then divided up into 5 × 5 equally sized blocks (D). For each block a histogram of the uniform local binary patterns was created (E) and these were then concatenated into one long histogram (F). This then formed the input to the classification algorithm (a 1475 element feature vector that was used either as training or testing data; G-H).

#### 2.5.3 Alignment and masks

The eye and nose detection stages produced x-y coordinates for the left eye, right eye and nose. The effect of aligning the facial images based on these coordinates was compare to the classification accuracy with no alignment. The images were rotated and scaled based on the two eye coordinates alone or all three coordinates.

A binary mask was applied that removed the corners of the image. The same mask was used all images. This was aimed at reducing the effect of back-lighting (due to the shape of the macaque face back-lighting was predominantly a problem for the corners of the images).

#### 2.5.4 Classification algorithms

The classification accuracy of three different classification algorithms was tested. The algorithms used were nearest neighbor (NN; Ahonen et al., 2006), local discriminant analysis (LDA; Fratric and Ribaric, 2011) and support vector machines (SVM; using a one vs one approach for mulitple classes; Lian and Lu, 2006). For the LDA classifier a diagonal covariance matrix was used and the same covariance matrix was used for each class. For the SVM classifier a linear kernel was used with a cost parameter of 1 and a one-vs-one coding approach. A linear kernel was chosen rather than a radial basis function kernel as there were a large number of features.

The effect of reducing the dimensionality of the local binary pattern histogram vector using principal component analysis (Fratric and Ribaric, 2011) was explored for all three classification methods. The number of principle components was chosen such that it explained >95% of the variance. Both classification and PCA were implemented using the standard Matlab functions.

### 2.6 Social networks

To test a practical application of the face recognition techniques to social behavior studies three days of video (∼5 h per day) of one group of ten adult female macaques was scored for proximity interactions using both traditional manual scoring and the automated face recognition.

#### 2.6.1 Manual scoring of proximity

The videos were scored for identity by a trained human observer (who had experience both with identifying individuals in that group and in behavior observations) at one minute intervals using custom written Matlab software. At each interval all monkeys present at that interval were considered in proximity with each other (approximate area covered by video frame was 2 m × 2 m).

#### 2.6.2 Automated scoring of proximity

Face detection and recognition as detailed above was run on the videos at a rate of 2 frames per second. A pair of females were considered in proximity with each other if there were at least three detections of each female of the pair within a 1 min window.

#### 2.6.3 Comparison

The free social network analysis software Socprog (Whitehead, 2009) was used to compile association matrices from the manual and automated proximity data and to test for correlation between the two association matrices (Mantel test for matrix correlation; Mantel, 1967). Preferred associations (individuals that associated with each other more than chance) were identified for both manual and automated methods by permuting the identities within each sample 10,000 times and recalculating the association matrix (Bejder et al., 1998). Associations were considered significant if the association matrix was greater than the 9950th value of the sorted permuted data (two-tailed test; P < 0.01).

### 2.7 Online face recognition

A laptop (Intel i7–3540 M (3.00 GHz) processor; 16GB Ram; Windows 7) with an attached USB camera (Point Grey Firefly MV; 50 mm lens; standard definition; www.pointgrey.com) was used to capture and process images. The camera was set up on the same window as the camcorder in Section 2.2. This was tested on one group of 14 individuals for 3 h. The group consisted of one adult male (Star), seven adult females (Linz, Maj, Mindy, Umbrella, Venus, Verity and Wine) and six infants aged 6–18 months. As it was difficult for a human observer to reliably identify the six infants, images from all six infants were combined to form an “infant” class. A face recognition model for that group was created from 100 images of each individual/class. Matlab, in combination with Point Grey software, was used to capture a frame; this was then processed using face detection (Section 2.4.2) and each detected face was classified using the face recognition model (Section 2.5). The processed frame was saved as a jpeg image so that the classification accuracy could be calculated. This was repeated at as high a frame rate as the software and hardware would support. At the end of the session the average frame rate and classification accuracy were calculated (classification accuracy was calculated by manually sorting the images offline).

### 2.8 Metrics

For face detection the two metrics used are sensitivity (% of actual faces successfully detected) and false positive rate (number of false positives as a % of total number of images). For face recognition the metric used is classification accuracy (% of faces correctly identified). This corresponds to the rank one metric often used in face recognition. Where possible multiple face recognition models were trained based on different training image sets randomly selected from the overall image sets. Unless explicitly stated the classification accuracy was calculated based on a separate testing set of 10 images per individual. For a small number of cases (made clear in the Results section) ten-fold cross-validation was used to provide a comparable measure to previous publications. The classification accuracy was calculated for each model and the results given as the mean ± the standard deviation.

### 2.9 Software

All analyses were carried out in Matlab (www.mathworks.com). The Matlab programs for face detection and recognition, the xml cascade classifier files, the face detection training image set and face recognition image set are available on Github (https://github.com/clwitham/MacaqueFaces).

## 3 Results

### 3.1 Face detection

The face detector was trained on images from the detection training image set and tested on 428 stills and images from the detection testing image set with known numbers and locations of faces. Fig. 1E shows the receiver operating characteristic curve for the face detector alone (face‐only detector; solid line) and face detector in combination with the eye and nose detectors (combination detector; dotted line). At low threshold values the face detector alone had a higher sensitivity (true positive rate) than the combination detector but at the cost of a much higher false positive rate (given here as the average number of false positives per image). When I raised the threshold of the detectors to reduce false positive rate to zero the combination method had a higher sensitivity (70%) than the face only method (60%). Fig. 1D shows an example of a processed video still with five faces detected using the combination method. Of the over 100,000 faces detected and sorted to form the recognition image set only two images were not real faces.

### 3.2 Face recognition

**Fig. 3:**
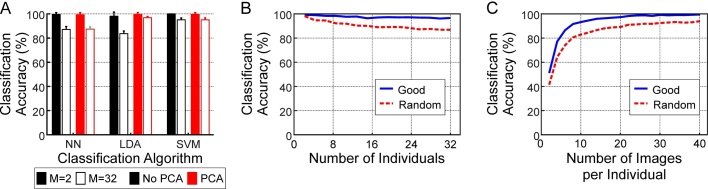
Success of face recognition. A, effect of classification algorithm on classification accuracy. Results given as mean ± standard deviation. B, effect of increasing number of individual monkeys on classification accuracy. Results given as mean only as standard deviations too small relative to plot size to show effectively. C, effect of increasing number of sample faces per monkey in the training set on classification accuracy. Results given as mean only as standard deviations too small relative to plot size to show effectively.

The recognition image set was used to test the classification accuracy of the face recognition protocol. First the classification accuracy of three different classification algorithms (NN, LDA and SVM) was tested with either the original LBP feature vector (1475 features) or with the PCA reduced vectors (375 features; principle components chosen such that they explained 95% variance across the dataset). For the testing each classifier was trained on a subset of the monkeys and well-aligned images. M individuals were randomly selected from the total of 34 monkeys (M ranged from 2 to 32) and N well-aligned face images randomly selected for each monkey (N ranged from 2 to 40). The classifier was then trained using the local binary pattern histograms as illustrated in Fig. 2H. The classification accuracy was calculated by using the trained classifier to predict the identity of a further 10 well-aligned images per individual (for a total of 10*M images per parameter combination). This was then repeated 10 times for each parameter using different combinations and the average classification accuracy taken. A subset of these results is shown in Fig. 3A. For very small numbers of monkeys (e.g. pairs) the classification accuracy of the three classifiers was comparable (Fig. 3A, black bars; classification accuracy shown for 20 images per individual). As the number of monkeys increased the classification accuracy of all three algorithms decreased but the LDA + PCA algorithm consistently produced the highest classification accuracy (Fig. 3A, white bars; 32 monkeys with 20 images per individual used for training). Reducing the dimensionality of the feature vector boosted the performance of the LDA classifier (open bars vs. solid bars) but had little effect on the performance of the other two classifiers. Table 1 shows the time taken for training and testing the different classifiers for the data shown in Fig. 3A. The NN algorithm was much quicker to train than the LDA and SVM algorithms. The LDA and NN algorithms had similar times for testing for both a set of 160 images and for a single image. The SVM algorithm was slower throughout. PCA reduced the training time for all three classifiers.

**Table 1:** Time taken for training and testing the different classifiers. Time for a single image includes time taken for extracting LBP features, reducing dimensionality (where PCA is used) and classifying the feature vector.

Classifier	NN	NN + PCA	LDA	LDA + PCA	SVM	SVM + PCA
Training Time (s) for 320 images	0.082	0.041	0.370	0.163	3.254	2.503
Testing Time (s) for 160 images	0.523	0.053	0.281	0.088	0.545	0.531
Testing time (s) for a single image	0.013	0.019	0.023	0.011	0.135	0.130

As the LDA approach gave the highest classification accuracy this approach was used to assess the effects of varying the number of individuals and the number of images per individual on the successful classification of the well-aligned (“Good” in Fig. 3B and C) and random (“Random” in Fig. 3B and C) faces. For the well-aligned images the classification accuracy remained >95% as the number of monkeys increased from 2 to 32 (Fig. 3B, solid line; 20 images per individual used for training; standard deviations not shown as too small relative to plot size but average standard deviation was 1.3%). For the randomly selected images, which included the images that were partially obscured, rotated or poorly lit, the classification accuracy dropped to 85% ± 1.3 (mean ± standard deviation) for 32 monkeys (Fig. 3B, dotted line). The number of images per individual used for training had a large effect on the classification accuracy of both well-aligned images and random images (Fig. 3C; 16 individuals used for training; standard deviations not shown as too small relative to plot size but average standard deviation was 1.9%). Low numbers of images (less than 8 images per individual) were particularly poor. As the numbers of images per individual increased the classification accuracy improved more slowly and between 20 and 40 images there was only a small improvement in classification accuracy.

**Fig. 4:**
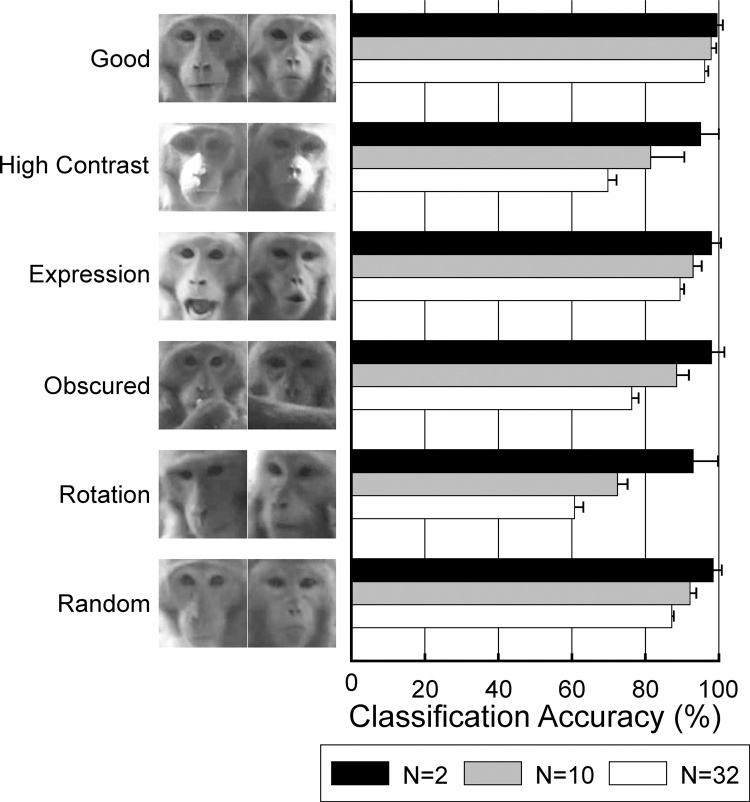
Effect of challenging image conditions on classification accuracy. Results given as mean ± standard deviation. N represents number of individuals used in training.

In a further test the different conditions that might affect the classification accuracy of the face recognition were investigated (Fig. 4). These categories were high contrast images due to sunlight (“high contrast”), poorly aligned images due to rotation of head (“rotation”), partially obscured images (“obscured”) and images with facial expressions (“expression”). Of these four categories “rotation” had the most detrimental effect, reducing the classification accuracy to 60.4% ± 2.4% for 32 monkeys (results given as mean ± standard deviation). “High contrast” also had a detrimental effect on classification accuracy (69.4% ± 2.5% for 32 monkeys). “Expression” and “obscured” images had higher classification accuracies (88.5% ± 2.0% and 76.2% ± 1.8% respectively).

As rotation had a detrimental effect on classification accuracy the effect of aligning the images through rotation and scaling based on the coordinates of the eyes only or the eyes and nose was investigated. Aligning the faces using either method actually reduced classification accuracy (for 32 monkeys with 20 images per individual the classification accuracy dropped from 85% ± 1.3% for unaligned images to 83.3% ± 2.1% for aligned images).

To give a better idea of overall performance in practice I divided the individuals into their respective groups (total of four groups) and used ten-fold cross-validation to calculate the classification accuracy for each group using the random image set. Group DA contained 1 male and 10 females, group JU contained 1 male and 5 females, group SO contained 1 male and 8 females and group ST contained 1 male and 7 females. The classification accuracy ranged from 90.5% for group DA to 96.2% for group SO (based on using 40 images per individual).

### 3.3 Application to social networks

I tested a possible application of face recognition to animal social analysis. Three days of video of one group of ten adult females was processed using the face detection and recognition protocols to produce a matrix based on proximity associations. All faces produced by the detection were also sorted manually by identity to give the classification accuracy and allow the impact of classification accuracy on association measures to be assessed. A total of 10013 facial images were produced across the three days of videos and the classification accuracy was 96.7%. To validate the proximity association measures the videos were also scored using traditional coding methods (scan sampling at 1 min intervals) and a second association matrix produced based on the manually scored data.

**Fig. 5:**
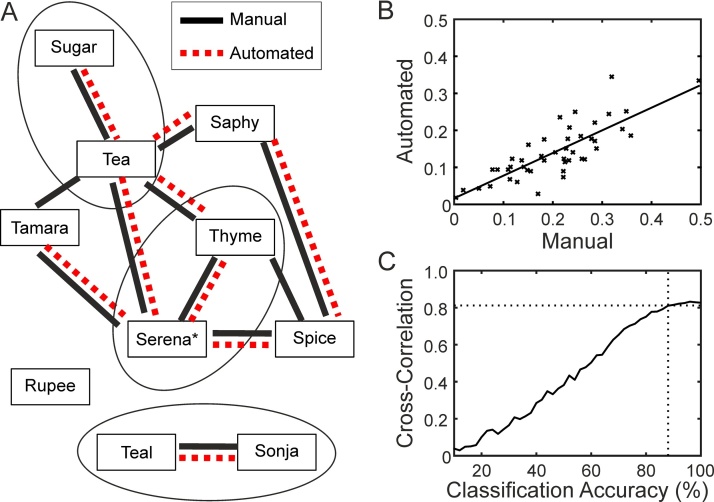
Face recognition and social analysis. A, significant preferred associations based on manual and automated analysis of videos of 10 adult female macaques. Pairs of full sisters are identified by ovals and the * symbol indicates the alpha female. B, comparison of association indices produced by the manual method (x-axis) and automated method (y-axis). Solid line indicated best-fit (linear regression). C, effect of classification accuracy on correlation between the association matrices produced by manual and automated scoring. Dotted lines indicate drop-off in correlation.

The significant preferred associations in this matrix are shown in Fig. 5A, with the results of the manual scoring given by solid lines and the results of automated scoring by dotted lines. This group contains 3 pairs of full sisters indicated by circles and the alpha female (highest ranked female) is indicated by an asterisk. As might be expected all three pairs of full-sisters have significant association and the alpha female (“Serena”) has a high number of associations with other females. One monkey “Rupee” has no significant preferred associations. At the time these videos were recorded she was the lowest ranked female in the group.

Fig. 5B shows the relationship between the association indices produced by manual scoring (x-axis) and automated scoring (y-axis). There is a clear linear relationship but the automated method underestimated the association index in comparison to the manual methods. This is also seen in Fig. 5A where the automated method produced two fewer significant associations than the manual method (these were Tamara-Tea and Thyme-Spice). The association matrix produced by the automated analysis was significantly correlated with the association matrix produced by manual analysis (Mantel Test; matrix correlation of 0.807; P < 0.001; analyzed using Socprog).

The effect of classification accuracy on the association values is shown in Fig. 5C. Starting with the ground-truth identity set I randomly assigned between 0 and 90% of images to other individuals. This artificially produced datasets with classification accuracy ranging from 10% (chance level for a group of 10) to 100%. I then recalculated the association matrix for each set and the matrix cross-correlation coefficients. This was repeated 10 times for each classification accuracy (with a different random set each time) and the average cross-correlation coefficient calculated. Fig. 5C shows these average cross-correlation coefficients plotted against classification accuracy. For the “perfect” dataset (classification accuracy of 100%) the cross-correlation coefficient was 0.820. This remained at similar levels until the classification accuracy dropped below 88% (indicated by dotted lines) whereupon the cross-correlation coefficients declined steadily down to zero.

### 3.4 Online face recognition

Online face recognition was tested on one group consisting of one adult male, 7 adult females and 6 infants (age range 6–18 months). As it was difficult to manually sort the infant images according to identity all infants were grouped together and classified as infants. An average frame rate of 15.8 frames per second was achieved and the classification accuracy was 95.1% (this was achieved by using a 100 images per individuals/class, which is a large number). Using a more restricted training set (35 images per class) reduced the overall classification accuracy to 88.0%. Table 2 shows the confusion matrix for this session.

**Table 2:** Confusion matrix for online face recognition. Numbers given as numbers of images with actual identity (given by row) and predicted identity (given by column). Actual identity determined by manual sorting of images. Final column shows % of images of that individual that were correctly classified.

		Predicted Identity									% Correct
		Star	Linz	Maj	Mindy	Umbre	Venus	Verity	Wine	Infant	
Actual Identity	Star	161	0	0	0	0	0	0	0	0	100.0
	Linz	0	77	0	9	1	0	0	0	1	87.5
	Maj	0	0	304	1	4	1	0	0	0	98.1
	Mindy	0	1	0	230	1	0	0	1	0	98.7
	Umbre	0	0	0	6	285	0	1	1	0	97.3
	Venus	0	10	0	8	11	275	1	10	1	87.0
	Verity	0	0	0	0	0	0	195	3	0	98.5
	Wine	0	1	1	3	1	0	3	88	1	89.8
	Infant	2	0	0	0	2	0	0	2	108	94.7

The adult male (Star) had 100% of his images correctly classified. Classification accuracy was lowest for Linz (87.5%), Venus (87.0%) and Wine (89.8%). Linz is the mother of Venus and Wine (the other mother-daughter pair in this group was Maj and Verity; these had a much higher classification accuracies of 98.1% and 98.5% respectively).

## 4 Discussion

In this study I have shown, for the first time, that it is possible to use automated face recognition to identify individual rhesus macaques with a classification accuracy of over 95% for well‐aligned (“good”) images. This can be done using basic hardware and can be implemented in real time. As might be expected the classification accuracy was highest when the face recognition model was trained on small groups of monkeys and high numbers of images per individual. However between 10 and 20 images per monkey produced reasonable classification accuracies for up to 32 monkeys.

### 4.1 Performance of different classification algorithms

For this setup a linear discriminant analysis classifier in combination with dimensionality reduction through principle component analysis produced the best results. The increased performance of the LDA classifier following dimensionality reduction is probably due to issues estimating the covariance matrix with the full feature vector. SVM performed almost as well as the LDA classifier and it is possible with more tuning of parameters (such as the cost parameter) that the performance could be improved. However the LDA classifier was quicker to train and so is still preferable (Table 1). The poorer performance of the nearest neighbor algorithm in this paper may be due to the large number of features (even after dimensionality reduction with PCA). Several papers based on human face recognition have also compared the performance of different classification algorithms. Pallabi and Bhavani (2006) showed that k-nearest neighbors in combination with PCA outperformed linear discriminant analysis and support vector machine classification for human faces. However they used the pixel intensity values as the input vectors rather than the local binary patterns used here.

### 4.2 Challenging conditions

Videoing under real world conditions produces a range of issues that can make face recognition challenging. During this study I have investigated several of the major issues: contrast, facial expressions, partial obscuration of face and rotations of face. Under these conditions the overall classification accuracy was reduced to 89% (for 20 individuals and trained on 20 images per individual). Of the four conditions facial expressions only had a small impact on classification accuracy compared to the well-aligned images. Rotation and contrast had much greater effects on accuracy. A similar study in chimpanzees encountered similar problems (Loos, 2012). An attempt was made to improve classification accuracy by aligning the images based on eye coordinates (Section 3.2). However this actually reduced classification accuracy. This may be due to the majority of rotations being out-of-plane (as shown in the examples in Fig. 4) and therefore not easily corrected by rotating the image. In future either these type of images should be automatically excluded or the face recognizer made more robust. Face recognition methods are rapidly advancing and new techniques should make it easier to overcome some of these issues.

Studies have shown that monkeys are capable of recognizing faces as belonging to kin rather than non-kin suggesting a certain level of facial similarity between closely related monkeys (Parr et al., 2010, Pfefferle et al., 2014). This could adversely affect the classification accuracy. Although not investigated here the model set did include a number of closely related individuals (mothers and daughters and full- and half-sisters) and the classification accuracy reported here includes these close relations Therefore we might expect the classification accuracy to be higher for a group of less related monkeys.

### 4.3 Uses

This study was initiated with the aim of producing a non-invasive method of identification that could be combined with tracking and behavior paradigms to monitor macaque behavior. This combination will be the focus of future work. However there are a number of potential uses that the face detection and recognition methods described here could be used for at this time. I have demonstrated one of these potential uses with a brief look at using face recognition to monitor proximity-based social associations between animals. This generally produced good agreement with the traditionally scored associations but consistently underestimated the association index. This is most likely due that unlike the automated face recognition method the observer carrying out the traditional scorer was able to identify monkeys even when they were facing away from the camera. It is likely by combining the face recognition with some form of tracking will boost the correlation with the traditional method. Another potential problem is how classification accuracy affects the validity of this approach. I showed in Fig. 5C that classification accuracies in the range 88–100% had little effect on the correlation with the traditional method. Most of the classification accuracies reported in this paper fall within that range. The automated system has the potential to save time over manual analysis. It takes 1–2 days to collect enough video to train the recognition model (with about 1–2 h of staff time to setup cameras and sort faces, it requires a member of staff who is familiar with the group but does not need to be trained in behavior observations). The system can be left running for 60–90 days without updating (from personal observations). In contrast the manual analysis takes an experienced member of staff 1–2 h per day to analyze the videos using the point sampling method described here (the member of staff has to be both trained in behavior observations and familiar with the group).

There are two other potential uses with applications to neuroscience research. Images and videos of macaques and other primates are used in neuroscience research as stimulus sets (see Fisher and Freiwald, 2015 for an example). Faces are one of the stimuli of interest. The face detection methods here can be used to automatically process these videos for location of faces saving the need for labor intensive manual scoring of the videos or to pull out clips of faces from much longer video sequences.

Another potential use, using both face detection and recognition methods, is as means of identifying which macaque is interacting with a home-cage based task. Automated home cage training systems are starting to be used both for early training of monkeys and in combination with freely moving recording devices (Calapai et al., 2016, Mavoori et al., 2005). Currently either RFID tags are used to identify individuals within a group (Fagot and Paleressompoulle, 2009; but some instituions have had issues with reliability; personal communication) or the monkeys have had to be separated off from the rest of the group. Face recognition could be used, either alone or in combination with RFID tags, to improve identification. For this the face detection and recognition methods must work in real time and be very effective with pairs or small groups of monkeys. Both of these have been demonstrated in this paper. However one potential complication is that due to the bias towards female monkeys in a breeding colony the model image set used in this paper contained many more females than males. In many neuroscience primate facilities the monkeys are more likely to be male than female. There is no particular reason why male faces should be less individually identifiable than female faces (if anything the opposite is likely to be true as males develop large canines and much stronger facial muscles than females) but this would need to be investigated.

### 4.4 Conclusions

I have shown that it is possible to automatically identify individual macaques by their faces in videos. This can be done in real time and the methods can be used at this time for a range of applications. Future work will include improving the classification accuracy especially under challenging conditions, combining face recognition with tracking to monitor behavior and to investigate other applications of these methods.
